# Research involving adults lacking capacity to consent: the impact of research regulation on ‘evidence *biased’* medicine

**DOI:** 10.1186/s12910-016-0138-9

**Published:** 2016-09-08

**Authors:** Victoria Shepherd

**Affiliations:** South East Wales Trials Unit, Centre for Trials Research, College of Biomedical and Life Sciences, Cardiff University, Room 406, 4th Floor, Neuadd Meirionnydd, Heath Park, Cardiff, CF14 4YS UK

## Abstract

**Background:**

Society is failing in its moral obligation to improve the standard of healthcare provided to vulnerable populations, such as people who lack decision making capacity, by a misguided paternalism that seeks to protect them by excluding them from medical research. Uncertainties surround the basis on which decisions about research participation is made under dual regulatory regimes, which adds further complexity. Vulnerable individuals’ exclusion from research as a result of such regulation risks condemning such populations to poor quality care as a result of ‘evidence *biased*’ medicine.

**Main Text:**

This paper explores the research regulation provisions for proxy decision making for those unable to provide informed consent for themselves, and the subsequent legal and practical difficulties for decision-makers. There are two separate regulatory regimes governing research involving adults who lack capacity to consent in England and Wales. The Mental Capacity Act 2005 governs how incapacitated adults can be involved in research, however clinical trials of medicinal products are separately regulated by the Medicines for Human Use (Clinical Trials) Regulations 2004. There are significant differences under these dual regimes in the provisions for those lacking capacity to participate in medical research. The level of risk permitted differs, with a greater requirement for justification for participation in a clinical trial than other types of research. Who acts as proxy decision maker, how much information is provided to the person lacking capacity, and whether they retain the power of veto also significantly differs.

**Conclusion:**

The development of two separate regulatory regimes has resulted in significant differences between the provisions for clinical trials and other forms of research, and from usual medical practice. The resulting uncertainty has reinforced the tendency of those approving and conducting research to exclude adults lacking capacity to avoid difficult decisions about seeking consent for their participation. Future developments, such as the incoming EU Regulations, may address some of these differences, however the justification and level of risk permitted requires review to ensure that requirements are appropriate and proportionate to the burdens and risks for the individual, and also to the benefits for the wider population represented.

## Background

Vulnerable populations in society, such as adults lacking mental capacity to make decisions for themselves, are routinely denied the opportunity to participate in health-related research [1–3]. Individuals may lack capacity as a result of a range of cognitive disorders or conditions, which may result in the inability of individuals to protect themselves by freely given informed consent. It has recently been proposed that there are two central, normatively opposed, ethical and legal problems relating to research involving vulnerable populations: that those unable to provide consent are entitled to special protection as they are unable to adequately protect themselves from exploitation; however if all those not having capacity to consent were excluded from research then medicines and other health interventions would remain untested in the target population [2]. Vulnerable groups are entitled to special protection in medical research as, historically, appalling ethical transgressions have resulted from the pursuit of scientific data which have often involved vulnerable individuals [4].

However concerns regarding the ethical and legal issues of informed consent by people who lack decision-making capacity as a result of cognitive impairment have been identified as barriers to participation in medical research [1, 5, 6]. Leading to claims that society is failing in its moral obligation to improve the standard of evidence-based healthcare provided to vulnerable populations, who are currently substantially disadvantaged by the tendency by research regulatory frameworks (and those that implement them) to protect them by excluding them from medical research [2, 4, 7]. Honouring this obligation requires a shift towards offering better protection by including them in more research projects, thereby allowing vulnerable populations to benefit from medical progress.

There have been recent developments to address the exclusion of some vulnerable groups. The European Medicines Agency, describing ‘evidence *biased* medicine’ as a global issue, has developed a Geriatric Medicines Strategy to ensure the availability of safe and effective medicines for an ageing population [8]. However, there have been calls for a more fundamental step change from the current legal and ethical position of ‘protection by exclusion’ to ‘protection by inclusion’, through shifting the emphasis of clinical research to the benefit of vulnerable populations [2].

Adults considered vulnerable require different decision-making processes for participating in a clinical trial than decisions made in usual medical care, and also regarding participation in other types of research. There are two separate regulatory regimes governing research involving adults who lack capacity to consent in England and Wales. The Mental Capacity Act (MCA) 2005 governs how incapacitated adults can be involved in research [9], though it excludes clinical trials of investigational medicinal products (CTIMPs). Clinical trials involving adults who lack capacity are separately regulated by the Medicines for Human Use (Clinical Trials) Regulations 2004 (CTR) [10]. Although there are some similarities between the two regimes, there are significant differences. Research projects are classified as CTIMP or non-CTIMP studies–with some larger research programmes incorporating both, such as a pre-clinical trial observational stage to ascertain prevalence of a particular condition, followed by a clinical trial to evaluate a proposed medical treatment or regime. The same research site, treating clinicians and participants may be involved in both stages but different regulatory frameworks, and hence different decision making processes, will apply.

This special treatment presents difficulties for those involved in the day to day care of adults lacking capacity who are familiar with the processes for making treatment decisions with this population, but are concerned about decisions regarding their participation in research [7]. The complexity of the current legal framework, its legislative differences, and uncertainty surrounding their interpretation, has resulted in confusion both for researchers and Research Ethics Committees [11], as well as clinicians, relatives, and carers involved in decisions about people participating in research [7]. This issue remains one to be addressed and, unless it is done so, strategies introduced to improve the inclusion of vulnerable adults in medical research may fail. This will perpetuate the current inequitable position where, for example, older people receive poorer levels of care than younger people with the same conditions [12]. Even within the older adult population, there is significant aged heterogeneity in health status from the relatively fit to the most frail, which limits the generalisation of already complex prescribing decisions [13]. The most vulnerable and frail have limited physiological reserve, reduced homoeostasis, dysregulations in immune and inflammation mechanisms, multiple comorbidities, and polypharmacy [14]. Further robust data about the risks and benefits of drugs in such populations–particularly the vulnerable and frail-is urgently needed [15].

This paper explores the differences between the two regulatory regimes and its impact on the exclusion of adults who may lack decision-making capacity from medical research participation.

## Discussion

### Scientific necessity for inclusion

The scientific validity of research cannot be justified if the expected benefits are extrapolated from the data obtained from other populations as differences between populations such as differences in physiology, pharmacokinetics and treatment responsiveness can be significant [16]. The importance of inclusion in medical research is a feature of national health research strategies and European and International initiatives [17], including the new EU clinical trials regulation which emphasises the importance of trial relevance [18] and comes into application in 2018. The new Regulation states that, unless otherwise justified in the research protocol, the subjects participating in a clinical trial should represent the population groups, for example gender and age groups, that are likely to use the medicinal product investigated in the clinical trial [18]. The Regulation also states that, currently, many trials are carried out in patient populations which do not necessarily reflect the diversity of the population group on which the drug will ultimately be used [18].

### Inequity of inappropriate exclusion

Those unable to give informed consent for themselves are often excluded from participating as a result of misguided paternalism and a desire to protect them from potential harms [19]. Concerns about the inclusion of vulnerable people are characterised by the much-cited views of Hans Jonas in the 1960’s that ‘The afflicted should not be called upon to bear additional burden and risk [involved in medical research], …they are society’s special trust and the physician’s particular trust-these are elementary responses of our moral sense’ [20]. However it can be argued that the very nature of their affliction places an increased imperative on the medical community and the wider society to search for ways of improving their plight.

Participation in research can sometimes bring direct benefits for participants, in terms of enhanced monitoring of their health or access to treatment not widely available outside a research programme. Excluding such groups, who may already experience health inequalities, from participating in research denies them the benefits that participation can bring and unfairly discriminates against them [21]. As the inverse research law proposes, those in the greatest need of research attention are those least likely to be the subject of research. To deny groups the benefits of medical research by unduly cautious regulation is considered to be ‘neither kind not caring but irresponsible’ by condemning such populations to poor quality care [22]. Kopelman goes further, claiming that the practice of routinely excluding vulnerable groups from research as a result of such regulation is literally ‘protecting them to death’ [23].

### Current research regulation

Adults are presumed to have capacity to make autonomous decisions about themselves unless proven otherwise [9]. The uncertainty regarding the legality of the inclusion of individuals lacking capacity has been clarified to some extent by the enactment of the MCA, which has particular provisions relating to research involving such individuals, although only to research that is not a clinical trial of a medical product [10]. These are regulated separately under the Clinical Trials Directive [24] and implemented in the UK by the Clinical Trials Regulations (CTR) [10]. However, there are significant differences under these two separate regulatory regimes in the provisions for those lacking capacity to participate in medical research (Table 1). These differences increase the burden on researchers and clinicians responsible for recruiting eligible participants which presents additional barriers to conducting research with vulnerable groups [25].

**Table 1:** Differences between provisions for adults lacking capacity under Mental Capacity Act 2005 (MCA) and Medicines for Human Use (Clinical Trials) Regulations 2004 (CTR)

	MCA	CTR
Who acts as decision maker	Researcher must consult: Personal consultee-a person who is engaged in caring for the person or is interested in their welfare, except as a professional or for remuneration–friend, relative, unpaid carer, attorney acting under LPA, court appointed deputy Nominated consultee-a person who has no connection with the project-healthcare professional, nominated individual	Legal representative is a person who, by virtue of their relationship, is suitable to act as their legal representative and is available and willing to act. Personal legal representative–friend, relative. Professional legal representative–the doctor primarily responsible for their medical treatment or a person nominated by their health care provider. Must not be connected with the trial.
Basis for the decision	Consultee asked for advice whether the participant should take part or would not have wished to participate. Responsibility whether to include the participant lies with the researcher	Informed consent given by the legal representative represents their presumed will. Representative to decide whether the participant would have wanted to participate had they capacity to do so.
Provision of information	Does not specify any provisions that the person has to be informed about the research once they have been assessed as lacking capacity	Person lacking capacity must have received information about the trial, its risks and benefits, according to his or her capacity before they can be involved.
Dissent/objection	Weight is given to any refusal or dissent from the individual lacking capacity, even when the person has little or no ability to understand the situation. If the person indicates (in any way) that he wishes to be withdrawn from the project he *must be withdrawn* without delay.	The explicit wish of a subject who is capable of forming an opinion and assessing the information to refuse participation in, or to be withdrawn from, the clinical trial at any time *must be considered* by the investigator
Level of risk permitted	Research must be connected with an impairing condition in the functioning of the mind or brain affecting the person, or its treatment. There must be reasonable grounds for believing that the risk to the person is *negligible* and that anything done in relation to the person will not interfere with their freedom of action or privacy in a significant way or be unduly invasive or restrictive.	The clinical trial must relate directly to a life-threatening or debilitating condition clinical condition from which the person suffers. There must be grounds for expecting that administering the product will *produce a benefit to the person outweighing the risks or produce no risk at all*

### Justification for inclusion–‘minimal risk versus no risk’

Research Ethics Committees have a statutory responsibility for reviewing research proposals involving human subjects which are categorised as intrusive and, if satisfied that the project meets the criteria, will issue ethical approval. In order for adults lacking capacity to participate in non-clinical trial research it must be connected with an impairing condition in the functioning of the mind or brain affecting the person (or its treatment), which causes or contributes to the impairment [9]. This requirement has been particularly challenging in situations where the research may not be directly connected to the impairing condition, but the impairment may be a risk factor for the condition under investigation [1].

Where the research is a clinical trial, the inclusion of adults lacking capacity is permitted only if the risks and inconveniences have been weighed against the anticipated benefits for the individual trial subject and future patients and that the benefits justify the risks. The clinical trial must relate directly to a life-threatening or debilitating condition clinical condition from which the person suffers and there must be grounds for expecting that administering the product will produce a benefit to the person outweighing the risks or produce no risk at all [10].

There is therefore a greater requirement for justification for participation in a clinical trial than other types of research. This additional justification required can impact on the ability to conduct clinical trials with vulnerable populations [26] by focussing on the classification of the intervention alone, rather than a proportionate and appropriate risk-based assessment of the research design itself [27]. The requirement that it is expected that the person themselves will either benefit from the product outweighing the risks or produce no risk at all is one of the problematic requirements. In order for this to be met, there must be an expectation that the product being tested is either entirely without risk, which is unlikely for any treatment, or is better than the standard treatment the person would have received. However clinical trials, especially in populations such as these, are often urgently required because existing standard treatments are being used despite the lack of evidence of its risks or benefits for these populations. At present there is little known about how some drugs commonly used in legally competent adults, such as anti-psychotics and anti-convulsants, affect those who may lack competence. There may be significant differences between those with and without capacity and the risks and benefits may be different for each group [26]. An example is individuals with Down’s syndrome, which is associated with a significantly higher risk of developing Alzheimer’s disease, who may have an atypical response to anti-dementia medication compared to other individuals and may experience a difference in disease progression than the general population [22]. In such situations the safety and efficacy of medicines for these groups needs to be established through a clinical trial which may involve individuals who, despite all reasonable attempts to enable them to provide consent for themselves, are found to lack capacity.

The Declaration of Helsinki [17] does not stipulate a limit for the degree of risk that may be permitted, but does require that the research must be justified by its potential value for future care. The incoming EU Regulations [18], adopted on 14th April 2014 and expected to come into application by October 2018, reflect this position by requiring that the trial either confers a direct benefit to the incapacitated subject, outweighing the risks and burdens involved; or some benefit for the population represented by the incapacitated subject concerned if the clinical trial relates directly to the life-threatening or debilitating medical condition from which the subject suffers and such trial will pose only minimal risk or burden to the incapacitated subject concerned *when compared with the standard treatment* of their condition.

### Who can act as proxy decision maker

The MCA provides for another person to be consulted for advice before the individual lacking capacity is included in the research. If the researcher is unable to identify a personal consultee who has an unpaid or non-professional role in caring for the person, they must appoint a nominated consultee who may be a healthcare professional or a nominated individual working for a relevant organisation such as a local authority [9].

The consultee must be provided with the information about the research and is asked whether the participant would have declined to take part if he or she had capacity [9] and any indication that they would not have wished to participate must be respected. Responsibility for deciding whether to include a person lacking capacity lies ultimately with the researcher. All other decisions relating to medical treatment and care made on behalf of an adult who lacks capacity are covered by the MCA provisions, generally under ‘best interests’ processes. This results in uncertainty and reluctance when decisions regarding the participation of such individuals in research are being made.

In clinical trials, when the person is not capable of giving consent, informed consent is given by his or her legal representative [10]. However the provision of informed consent by proxy, rather than simply advice as stated under the MCA, has been considered as a landmark shift in medical law. Previously the position under the common law was that, even where an adult patient lacked the mental capacity (either temporarily or permanently) to give or withhold consent for themselves, no-one else could give consent on their behalf. This exceptional consent by proxy is problematic for health professionals involved in decision making but inexperienced in conducting research involving adults lacking capacity.

Thus the MCA gives the consultee a right of veto but not a right to give their consent, in contrast to the regulations for a clinical trial of a medicinal product where consent by proxy is given. These differences are not widely understood and can be a source of confusion amongst regular care givers for those lacking capacity who are familiar with other decision making processes, such as through best interests meetings.

### Basis for the decision

A core principle of the MCA is that all decisions and actions must be in the best interests of the person lacking capacity [9]. However, best interest decisions do not apply when considering the involvement of someone who lacks capacity in research, which involves a different process and criteria. The researchers must seek advice regarding whether the person should take part in the project, and what, in his opinion, their wishes and feelings about taking part in the project would be likely to be if they had capacity in relation to the matter [9]. Where the individual acting as a consultee has other moral, professional or legal obligations towards the person lacking capacity, they may have a prevailing obligation to protect their best interests. Therefore, in addition to establishing what the person’s wishes and feelings about taking part in the project would be likely to be if they had capacity, the basis for this proxy or surrogate decision-making may involve a consideration of their best interests. However ‘best interests’ has been argued to be an ethically weak basis for decision making in medical research for those lacking capacity as, whilst there may be future benefit from the medical knowledge obtained, there may no direct benefit to the participant [28]. Indeed, it has been suggested that medical research ‘can never be primarily in a patient’s best interests’ [29] but intended to benefit both future persons and the common good [28]. It is unclear how this best interests obligation can be balanced against any conflicting advice about the person’s wish to participate. The relative weight given to any assent or dissent/refusal from the person, balanced against any advance decision (a ‘living will’) previously made by the person, is unclear. Although nothing may be done to the person in the course of the research which would be contrary to any advance decisions by them which has effect [9].

In clinical trials, the legal representative must decide whether the person lacking capacity should participate in the trial on the basis of what they would have wanted had they the capacity to choose for themselves, their presumed will. They need only consider, not necessarily respect, the person’s actual wishes on the day, even if they dissent. This decision is not based on the person’s best interests and it is unclear how the legal representative can presume another’s will, given that there may be little or no evidence of their intent, without it being a form of substituted judgement. The adult lacking capacity may never have had decision making capacity at any point. These difficulties become even greater when the relationship between the person lacking capacity and the legal representative becomes more distant. The CTR provides that, where a personal representative cannot be found, a ‘professional’ representative may be sought by the researchers. This person, usually another doctor, may know nothing about the wishes of the patient concerned and may not have been involved in their care at a time when they had capacity.

The incoming EU Regulations has left a wide margin for the application of consent by proxy, by leaving the Member States to determine the legally designated representatives of incapacitated persons and minors [18].

### Requirement for provision of information

The level of information required to be provided to the person who lacks capacity is different for those involved in a clinical trial from those participating in other forms of research. Whilst the MCA does not specify any provisions that the person has to be informed about the research once they have been assessed as lacking capacity [9], the CTR requires that the person lacking capacity must have received information about the trial, its risks and benefits according to his or her capacity before they can be involved [10].

### Dissent or power of veto

There are differences in the weight given to any assent or dissent from the person lacking capacity between the legislation concerning clinical trials and that governing other forms of research. Under the MCA much weight is given to any refusal or dissent from the individual lacking capacity, even when the person has little or no ability to understand the situation. Section 33 of the MCA states that if the person indicates (in any way) that he wishes to be withdrawn from the project he must be withdrawn without delay [9]. It clearly indicates that any objection is considered a veto but does not specify any conditions, such as informing the patient according to their level of understanding, and is in sharp contrast to the provisions for clinical trials. Under the CTR the person unable to give informed consent must have received information about the trial according to his/her capacity. However the explicit wish of a subject capable of assessing this information and forming an opinion refuses to participate must only be *considered* by the investigators [10]. Whilst any appropriately informed dissent must be considered by the investigator, it does not have to be respected when ultimately deciding the matter. As a result, in a clinical trial a patient’s wishes need only be considered, however these wishes must at least be informed to some extent.

Thus the CTR appear to protect the person’s current wishes less vigorously than the MCA, despite requiring the provision of more information and a more active endorsement on the part of the subject [30]. Despite the emphasis on the provision of information to the person lacking capacity, there is no requirement to seek the agreement of the person; assent is considered to be the mere absence of an objection.

### Addressing the imbalance

The requirement for a clinical trial to relate directly to a life-threatening or debilitating condition clinical condition from which the person lacking capacity suffers is particularly problematic. Adults lacking capacity may be at high risk of developing conditions as a result of the physiological frailty and vulnerability that may accompany cognitive impairment, but may not currently suffer from the condition under investigation. Additionally, the condition being investigated may not ‘cause or contribute to’ the impairing condition, but may be a consequence of that condition, such as aspiration pneumonia. Commonly, new interventions are sought to reduce the risk of vulnerable individuals developing such conditions. If the proposed intervention is a medicinal product there must be grounds for expecting that administering the product will produce a benefit to the person outweighing the risks or produce no risk at all [10], which may be problematic in placebo-controlled randomised trials. However the benefits and risks cannot be ascertained without rigorous clinical trials in the population most at risk of the condition. The benefits and risks in a healthier cohort, even of similar age profile, will materially differ. Existing treatments and interventions, and indeed the condition itself, will already pose accompanying risks. The risks which may accompany research participation need to be balanced against the harms which may result from the use of existing treatment which has not been properly evaluated in the practice population. Non-CTIMPs already permit a ‘negligible’ risk to the person [10], and incoming clinical trial regulations allow a minimal risk or burden *when compared to the standard treatment* for their condition [20]. It has also introduced the concept that such risks may be justified by the potential value for the future care of the wider population the person lacking capacity represents, in line with the Declaration of Helsinki [17].

There is an opportunity for those responsible for research approval and governance, as well as clinicians, relatives, and carers involved in decisions about people participating in research, to review whether the interests of people who lack capacity are best served by excluding them from the improvements in care that only research can demonstrate are safe and effective. The level of risk permitted for all types of research, the requirement for provision of information, and the power of veto or dissent however uninformed should be more closely aligned in order to harmonise and simplify processes and therefore address the current imbalance.

## Conclusions

Medical research is essential to improve healthcare for all members of society. Those individuals or groups in society who are considered vulnerable must have equitable access and opportunity to participate in research as all other groups. Actively seeking the involvement of marginalised groups upholds ethical principles by ensuring that the benefits of research are distributed widely across society, and by seeking to improve health care both for the individual concerned and the group they represent. These groups already experience health disparities, which will persist indefinitely unless research into the conditions and specific treatments that affect these individuals is supported. Vulnerable people commonly have complex health care needs, but may experience poorer levels of care and higher levels of adverse effects from medication use than their cognisant counterparts. However involving vulnerable people in medical research, particularly those with cognitive impairment, is problematic.

Research involving vulnerable groups, such as adults lacking capacity, raises many ethical and legal issues: particularly with respect to informed consent for them to be included in a project. The current legal position is that adults lacking capacity are excluded from medical research unless their inclusion can be amply justified. However this position, rather than a requirement for their *exclusion* to be justified, conflicts with the moral obligation to improve evidence-based health care for all–particularly those with the highest health care needs and most vulnerable to adverse events.

Where an individual is unable to provide informed consent for themselves, alternative arrangements for proxy decision making exist. The development of two separate regulatory regimes has resulted in significant differences between the regulatory approaches to clinical trials and other forms of research, and from usual medical practice. The level of risk permitted differs, with a greater requirement for justification for participation in a clinical trial than other types of research. Who acts as proxy decision maker, how much information is provided to the person lacking capacity, and whether they retain the power of veto also significantly differs. As a result, there is uncertainty about the involvement of adults lacking capacity in different types of research, and the provisions and interpretation of different regulations. These complexities have led to uncertainty about their inclusion, which reinforces the tendency by those approving and conducting research to exclude them in order to avoid difficult decisions about seeking consent for their participation.

Future developments, such as the incoming EU Regulations, may address some of these differences, however at present adults lacking capacity are treated differently from other groups and experience inequality and less innovation in their medical care and treatment, as a result. In order to redress the current ageist ‘evidence bias’, the impact of research regulation must be addressed. The justification and level of risk permitted for participating in research requires review to ensure that requirements are appropriate and proportionate to the burdens and risks for the individual and also to the benefits for the wider population represented. Widespread exclusion from participation in research carries intrinsic risks for vulnerable groups, as they may be denied access to novel treatments or new preventative interventions, and existing treatments may remain largely untested in this population.

Further research is needed to explore caregivers’ attitudes and knowledge of the legal basis for decision-making, and to evaluate the impact of the current regulatory regimes on conducting research with vulnerable populations. The provision of information and any power of veto or dissent should be harmonised and simplified in order to redress the current imbalance. Comprehensive guidance is urgently needed for clinicians, relatives, and carers involved in decisions about people participating in research. Information about balancing best interests, presumed will and advance decisions is required to support decision-makers whilst adequately protecting vulnerable groups. Fundamentally, a shift must be made away from misguided protectionism, and towards promoting appropriate inclusion of adults lacking capacity in order to honour society’s obligation for equitable health care for all.

## Abbreviations

CTIMP: Clinical trial of an investigational medicinal product
CTR: Medicines for human use (Clinical trials) regulations 2004
MCA: Mental capacity Act 2005

## References

[1] Head MG, Walker SL, Nalabanda A, Bostock J, Cassell JA. Researching scabies outbreaks among people in residential care and lacking capacity to consent: A case study. Public Health Ethics. Advanced Access published online 16 Apr 2015. doi:10.1093/phe/phv011.

[2] Helmchen H, Hoppu K, Stock G, Thiele F, Vitiello B, Weimann A. From exclusion to inclusion: Improving clinical research in vulnerable populations. Berlin: Berlin-Brandenburg Academy of Sciences and Humanities. 2014. http://edoc.bbaw.de/volltexte/2014/2592/pdf/BBAW_Vulnerable_Populationen_PDFA1_b.pdf. Accessed 22 June 2015.

[3] Lewis K. Participation of people with a learning disability in medical research. Royal Mencap Society. 2014. https://www.mencap.org.uk/sites/default/files/documents/Participation%20of%20people%20with%20a%20learning%20disability%20in%20medical%20_1.pdf. Accessed 6 June 2016.

[4] Lott JP (2005). Vulnerable/special participant populations. Developing World Bioethics.

[5] Maas M, Kelle L, Park M, Specht J (2002). Issues in conducting research in nursing homes. West J Nurs Res.

[6] Hall S, Longhurst S, Higgingson I (2009). Challenges to conducting research with older people living in nursing homes. BMC Geriatr.

[7] Wood F, Prout H, Bayer A, Duncan D, Nuttall J, Hood K, Butler CC (2013). Consent, including advanced consent, of older adults to research in care homes: a qualitative study of stakeholders’ views in South Wales. Trials.

[8] European Medicines Agency. Geriatric Medicines Strategy. 2011. http://www.ema.europa.eu/docs/en_GB/document_library/Other/2011/02/WC500102291.pdf. Accessed 22 June 2015.

[9] Mental Capacity Act. London: Her Majesty's Stationery Office (HMSO). 2005. http://www.legislation.gov.uk/ukpga/2005/9/contents. Accessed 7 Sep 2016.

[10] Medicines for Human Use (Clinical Trials) Regulations 2004, SI 2004/1031. http://www.legislation.gov.uk/uksi/2004/1031/pdfs/uksi_20041031_en.pdf. Accessed 7 Sep 2016. 15812991

[11] Dixon-Woods M, Angell EL (2009). Research involving adults who lack capacity: how have research ethics committees interpreted the requirements?. J Med Ethics.

[12] Melzer D, Tavakoly B, Winder R, Richards S, Gericke C, Lang I (2012). Health care quality for an active later life: improving quality of prevention and treatment through information: England 2005 to 2012. A report from the Peninsula College of Medicine and Dentistry Ageing Research Group for Age UK.

[13] Nelson EA, Dannefer D (1992). Aged heterogeneity: fact or fiction? The fate of diversity in gerontological research. Gerontologist.

[14] Fried L, Walston JWJ, Hazzard WH, Blass JP, Halter JB (2003). Frailty and failure to thrive. Principles of geriatric medicine and gerontology (5th edn.).

[15] Spinewine A, Schmader KE, Barber N, Hughes C, Lapane KL, Swine C, Hanlon JT (2007). Appropriate prescribing in elderly people: how well can it be measured and optimised?. Lancet.

[16] European Forum Good Clinical Practice. Medical research for and with older people in Europe. 2013. http://www.efgcp.eu/Downloads/EFGCP%20GMWP%20Research%20Guidelines%20Final%20edited%202013-05-27.pdf. Accessed 22 June 2015.

[17] World Medical Association (2013). World Medical Association Declaration of Helsinki: ethical principles for medical research involving human subjects. JAMA.

[18] Council Regulation (No. 536/2014) (Clinical trials on medicinal products for human use) OJ L158.

[19] Sutton LB, Erlen JA, Glad J, Siminoff LA (2003). Recruiting vulnerable populations for research: revisiting the ethical issues. J Prof Nurs.

[20] Jonas H (1969). Philosophical Reflections on Experimenting with Human Subjects. Daedalus.

[21] Wrobel P, Dehlinger-Kremer M, Klingmann I (2011). Ethical Challenges in Clinical Research at Both Ends of Life. Drug Inf J.

[22] Liddell K, Bion J, Chamberlain D, Druml C, Kompanje E, Lemaire F (2006). Medical research involving incapacitated adults: implications of the EU clinical trials directive 2001/20/EC. Med Law Rev.

[23] Kopelman LM, Smith Iltis A (2006). When Should Research With Infants, Children, Or Adolescents Be Permitted?. Research Ethics.

[24] Council Directive 2001/20/EC (Clinical Trials Directive) OJ L121

[25] Whelan PJ, Walwyn R, Gaughran F, Macdonald A (2013). Impact of the demand for ‘proxy assent’ on recruitment to a randomised controlled trial of vaccination testing in care homes. J Med Ethics.

[26] Hood K, Nuttall J, Gillespie D, Shepherd V, Wood F, Duncan D, et al. Probiotics for Antibiotic-Associated Diarrhoea (PAAD): a prospective observational study of antibiotic-associated diarrhoea (including Clostridium difficile-associated diarrhoea) in care homes. Health Technol Assess. 2014;18(63):1. 10.3310/hta18630 PMC4781053 25331573

[27] Shepherd V, Nuttall J, Hood K, Butler CC (2015). Setting up a clinical trial in care homes: challenges encountered and recommendations for future research practice. BMC Res Notes.

[28] Berger JT (2011). Is best interests a relevant decision making standard for enrolling non-capacitated subjects into clinical research?. J Med Ethics.

[29] Corrigan OP, Williams-Jones B (2003). Consent is not enough—putting incompetent patients first in clinical trials. Lancet.

[30] Edwards SJL. Vulnerable adults in research: A tale of two regulations. Presented at London School of Economics 15/02/2007. http://www.lse.ac.uk/humanRights/aboutUs/articlesAndTranscripts/Testing_Medicines_Edwards.pdf. Accessed 22 June 2015.

